# Targetable Vulnerabilities in MYC‐Driven B Cell Lymphomas Resistant to BCR Extinction

**DOI:** 10.1002/hon.70175

**Published:** 2026-02-11

**Authors:** Silvia Brambillasca, Nicara Chantal Parr, Adriana Palmeri, Adrian Andronache, Hiroshi Arima, Giovanni Faga’, Brian Leuzzi, Laura Perucho, Michela Robusto, Maurizio Pasi, Federica Mainoldi, Daniele Fancelli, Mario Varasi, Gabriele Varano, Ciro Mercurio, Stefano Casola

**Affiliations:** ^1^ Experimental Therapeutics Program IFOM ETS‐The AIRC Institute of Molecular Oncology Milano Italy; ^2^ Genetics of B Cells and Lymphoma Unit IFOM ETS‐The AIRC Institute of Molecular Oncology Milano Italy; ^3^ Department of Medical Biotechnology and Translational Medicine University of Milano Milano Italy

**Keywords:** B cell receptor (BCR), BCR‐less lymphoma, CDK4/6, drug screening, high‐grade B cell lymphoma, mTOR, polatuzumab‐vedotin

## Abstract

Polatuzumab vedotin, an antibody‐drug conjugate (ADC) targeting the B cell receptor (BCR) signaling subunit CD79B, has recently entered frontline therapy for diffuse large B cell lymphoma (DLBCL) and high‐grade B cell lymphoma (HGBCL), achieving encouraging clinical results. However, MYC‐driven B cell lymphomas, particularly HGBCL with *MYC* and *BCL2* rearrangements, frequently silence surface BCR/CD79B expression, limiting the therapeutic reach of CD79B‐directed ADCs and underscoring the need for complementary treatment strategies. To uncover drug vulnerabilities associated with BCR extinction in aggressive B cell lymphomas, we conditionally ablated surface BCR expression in the *λ*‐MYC mouse B cell lymphoma model and screened 1475 small‐molecule compounds, including clinically approved agents, on syngenic BCR‐positive and BCR‐negative tumor cells. This screening revealed compounds with comparable activity across both states as well as compounds with improved efficacy against BCR‐deficient lymphomas. Notably, inhibitors of mTORC1/2 and CDK4/6 displayed robust and reproducible potency in BCR‐negative lymphoma cells. Mechanistically, BCR loss impaired mTOR‐dependent anabolic control, reducing protein synthesis, while diminished Cyclin D3 abundance sensitized tumor cells to pharmacological CDK4/6 blockade. Human BCR‐negative HGBCL with *MYC* and *BCL2* rearrangements similarly exhibited sub‐micromolar sensitivity to mTORC1/2 and CDK4/6 inhibitors. Overall, our results uncover targetable vulnerabilities in MYC‐driven B cell lymphomas, possibly extending to other aggressive B cell tumors silencing BCR expression. The data provide a rational basis for integrating CD79B‐directed ADCs with mTOR or CDK4/6 inhibitors to prevent or overcome treatment resistance of aggressive B cell lymphomas.

## 1 Introduction

Most mature B cell lymphomas share with their normal counterparts the surface expression of a B cell receptor (BCR) [1]. Beyond serving as a phenotypic marker, BCR signaling, either tonic or chronically active, is a major determinant in the genesis and persistence of several aggressive B cell malignancies, including Burkitt lymphoma (BL) and diffuse large B cell lymphoma (DLBCL) [2]. This dependency is further underscored by recurrent somatic mutations affecting structural or signaling components of the antigen receptor pathway [3]. The clinical significance of sustained BCR activity is illustrated by the remarkable efficacy of small‐molecule inhibitors targeting Bruton's tyrosine kinase (BTK), a proximal BCR effector, which have transformed the treatment of chronic lymphocytic leukemia and improved outcomes in mantle cell lymphoma, Waldenström macroglobulinemia, and activated B cell (ABC)‐type DLBCL [4, 5]. The ubiquitous expression of surface BCR on most mature B cell neoplasms has also provided a rationale for the development of antibody‐drug conjugates (ADC) that exploit the BCR subunit CD79B as a delivery vehicle for cytotoxic payloads [6]. Polatuzumab vedotin, an anti‐CD79 B ADC that couples a monoclonal antibody to monomethyl auristatin E (MMAE), a potent microtubule inhibitor, represents the first CD79B‐targeted ADC approved for frontline treatment of DLBCL and high‐grade B cell lymphoma (HGBCL), including the clinically aggressive subset harboring *MYC* and *BCL2* rearrangements (hereafter termed HGBCL‐DH‐*BCL2*) [7]. Both randomized and real‐world data indicate that pola‐R‐CHP significantly improves progression‐free survival (PFS) versus R‐CHOP in previously untreated DLBCL, whereas an overall survival benefit confirmed at the population level remains to be demonstrated [8, 9, 10]. Exploratory analyses suggest a greater PFS effect in non‐GCB‐type DLBCL [8, 9, 10]. Recent findings indicate that aggressive MYC‐driven B cell lymphomas, including HGBCL‐DH‐*BCL2*, spontaneously extinguish surface BCR expression in a substantial fraction of cases [11, 12]. Using MYC‐driven preclinical mouse and human lymphoma models, independent reports have shown that conditional inactivation of the BCR does not necessarily arrest tumor growth [13, 14]. Instead, MYC‐driven lymphomas display a striking degree of plasticity, rapidly adapting to the loss of BCR‐derived survival, proliferative, and metabolic cues [13, 14]. The recurrent identification of BCR‐negative variants among human MYC‐driven B cell lymphomas [11, 13] raises important therapeutic implications. Patients harboring such variants may experience suboptimal responses to pola‐R‐CHP therapy due to reduced or abrogated surface CD79 B expression [11]. Moreover, selective pressure exerted by CD79B‐targeted ADC could favor the outgrowth of BCR‐silenced clones, leading to acquired resistance. Understanding the adaptive mechanisms that enable MYC lymphoma cells to survive and proliferate without a functional BCR is therefore critical for identifying therapeutic vulnerabilities in BCR‐less malignancies.

We hypothesized that BCR extinction in MYC‐driven lymphomas induces compensatory signaling and metabolic adaptations that could be pharmacologically exploited. To test this, we performed a comparative in vitro screen of 1475 small‐molecule compounds, including clinically approved and investigational agents, using paired *λ*‐MYC murine lymphomas representing tumors before and after inducible BCR extinction. This approach enabled the identification of compounds retaining or gaining efficacy in the BCR‐deficient state. Collectively, our results identify a class of drugs with potent activity against BCR‐less MYC lymphomas, with selected hits validated in spontaneously arisen BCR‐deficient human HGBCL‐DH‐*BCL2*. These findings provide a therapeutic framework for targeting BCR‐independent variants of MYC‐driven B cell lymphomas, including those that may emerge under selective pressure from CD79B‐directed ADC therapies.

## 2 Methods and Materials

### 2.1 Mouse and Human MYC‐Driven Lymphoma Cell Line Models

Mouse *λ*‐MYC; B1‐8f lymphoma cell lines established from independent primary tumors were previously described [13], and grown at 2 × 10^5^ cells/mL in high‐glucose DMEM (Euroclone) supplemented with 10% heat‐inactivated FBS, 0.1 mmol/L non‐essential amino acids (Biowest), 1 mmol/L sodium pyruvate, 50 μmol/L *β*‐mercaptoethanol (Thermo Fisher Scientific), and 2 mmol/L L‐glutamine (Euroclone). HGBCL‐DH‐*BCL2* COH‐DHL1 cells [15] were cultured in RPMI‐1640 medium (Euroclone) supplemented with 10% heat‐inactivated FBS (Sigma‐Aldrich), 2 mmol/L L‐glutamine, and 10 mmol/L HEPES (Euroclone) at a density of 0.5–1.5 × 10^6^ cells/mL. The DoGKIT HGBCL‐DH‐*BCL2* cell line was cultured in RPMI 1640 supplemented with 10% heat inactivated FBS, 10 mmol/L HEPES (Euroclone) and 50 μmol/L *β*‐mercaptoethanol (Thermo Fisher Scientific) at a density of 0.2 − 1 × 10^6^ cells/mL. The WILL‐2 HGBCL‐DH‐*BCL2* cell line was purchased from DMSZ and cultured according to instructions reported by the biorepository. Cell lines were periodically tested for *Mycoplasma* (MycoAlert *Mycoplasma* Detection Kit, Lonza) and grown at 37°C in a humidified atmosphere with 5% CO_2_. Cell line authentication was performed using the GenePrint 10 System (10‐Locus STR System for Cell Line Authentication, Promega).

### 2.2 TAT‐Cre Transduction

TAT‐Cre transduction of *λ*‐MYC; B1‐8f lymphoma cells was performed as previously described [13]. Briefly, lymphoma B cells were resuspended in serum‐free medium (Hyclone) at 5 × 10^6^ cells/mL and transduced with 50 μg/mL recombinant TAT‐Cre protein, for 45 min at 37°C. Transduced cells were diluted in complete medium, washed and resuspended at 2 × 10^5^/mL to allow cell recovery and expansion. Frequency of surface IgM/BCR‐deleted cells was determined by flow cytometry 36 h after transduction. BCR‐less cells were purified by magnetic activated cell sorting (MACS) using a depletion protocol based on biotin‐labeled anti‐mouse IgM (Table S4), followed by anti‐biotin beads (Miltenyi Biotech, Germany), or by fluorescence‐activated cell sorting (FACS) using an Aria cell sorter (BD Pharmingen, USA), after staining with fluorescently labeled anti‐IgM monovalent Fab fragments (Jackson ImmunoResearch).

### 2.3 Construction of the Drug Library

The compound library used for the screening consisted of 1475 small molecules sourced from Selleck Chemicals, including approved drugs both for oncological and non‐oncological indications, small molecules at different stages of preclinical and clinical development, and a small subset of compounds synthetized by our internal IFOM‐ETS medicinal chemistry laboratory. The screening run set (organized with 41 compounds per plate) was prepared by generating a five points dose‐response curve for each compound, with a 1:10 serial dilution starting from 10 mM, in dimethyl sulfoxide (DMSO; VWR Chemicals). The dilutions were prepared in a V‐bottom 384‐well polypropylene plates (Thermo Fisher Scientific), stored at 4°C, and subsequently diluted 1:8 in cell medium before adding to the cell plates.

### 2.4 High Throughput Drug Screening

Murine tumoral B cells of the *λ*‐MYC lymphoma line # 2646 were seeded in flat‐bottom white 384‐well plates (Greiner) at a density of 1 × 10^3^ cells/well in 35 μL of medium. The following day, cells were treated with 5 μL from the screening run set pre‐diluted 1:25 in complete medium (final drugs concentrations were 50–5–0.5–0.05–0.005 μM). In all assay plates, 0.5% DMSO and 0.1 μM Panobinostat (Selleck Chemicals) were used as negative and positive controls, respectively. After 24 h of incubation, cell viability was assessed with CellTiter‐Glo 2.0 (CTG; Promega) by addition of 40 μL CTG 1:1 in ddH2O, and luminescence was measured using the Enspire (Revvity) plate reader. All steps of cell seeding, treatment, and CTG addition were performed using a MicroLab StarM (Hamilton) liquid handling system. The screening was conducted in three rounds, resulting in a total of 72 assay plates processed. All viability data were managed by a custom data management system that fully integrates compound library management, sample tracking and data registration. Viability data associated with individual treatments on each cell line were subsequently processed through a standard processing and analysis pipeline implemented in Matlab (MathWorks). A first data preprocessing step ensured the removal of potential systematic spatial effects on each assay plate. For this, we estimated a correction plane by MLE regression interpolating the data of Negative Controls samples. Then, the data on each assay plate was normalized by estimating the Viability Percent (100% for negative controls and 0% for positive controls). A Quality Control (QC) test on every assay plate was further used to select data undergoing further statistical analysis (Z‐factor > 0.5 between Negative and Positive Controls data). Then, we estimated the Differential Viability Percent between the responses of each compound at each dose on each cell line (i.e., % Viability BCR^−^ ‐ % Viability BCR^+^). This was further normalized to a robust z‐score, which was estimated based on the median‐absolute‐deviations (MAD) of the Differential Viability Percent. Finally, candidate hit compounds were defined as those molecules that preferentially reduced viability in BCR^−^ cells (z‐score < −2) or BCR^+^ (z‐score > +2), and selected after visual inspection of the corresponding dose‐response curves. Additionally, Highly Active compounds were defined as those molecules that reduced cell viability by more than 65% at the 0.5 μM dose on both cell lines (BCR^−^ and BCR^+^).

### 2.5 Drug Validation in Mouse and Human MYC‐Driven B Cell Lymphomas

BCR^+^ and BCR^−^ tumor B cells from lymphomas # 2646 and # 2567 were mixed in equal numbers, plated at 1 × 10^5^ cells/mL and cultured in medium supplemented respectively with Rapamycin (5, 10 or 20 nM; LC laboratories), Sapanisertib (10, 15 or 30 nM; Selleck Chemicals), CHIR99021 (1 μM; Medchem) or Palbociclib (62.5, 125, or 250 nM; Medchem) for up to 72 h. Vehicle‐treated cells were used as control. Medium‐containing inhibitors was refreshed every second day. Frequency of BCR^+^ and BCR^−^ lymphoma cells was measured by flow cytometry after 48–72 h.

BCR‐less HGBCL‐DH‐*BCL2* human lymphoma cell lines DoGKIT, COH‐DHL1, and WILL‐2 [11], were seeded at a density of 6 × 10^4^ cells/mL (2 × 10^3^ cells/well in 35 μL), in a white, flat‐bottom 384‐well microplate (Greiner). The next day, cells were treated with 5 μL of 1:5 serial dilutions of inhibitors targeting mTOR (Rapamycin, Everolimus and Sapanisertib at final concentrations 30 μmol/L—0.015 nmol/L) or CDK4/6 inhibitors (Palbociclib, Ribociclib and Abemaciclib at final concentrations 30 μmol/L—0.015 nmol/L) for 72 h. At the end of the treatment, 40 μL CellTiter‐Glo 2.0 Cell Viability Assay substrate (CTG; Promega) diluted 1:1 in ddH2O, was added to each well, and incubated for 15 min. Luminescence signal was measured using the Enspire plate reader (Revvity). All treatments were performed in *n* = 3 biological replicates.

### 2.6 Puromycin Incorporation Studies

Protein synthesis was measured using the SUnSET method, as previously described [16]. BCR^+^ and BCR^−^ lymphoma B cells were seeded at 4 × 10^5^/mL in complete medium, pulsed with 10 μg/mL of Puromycin and incubated for 30 min at 37°C, 5% CO_2_. Cells pre‐treated with Cycloheximide (CHX, 10 μg/mL) were used as negative control. Puromycin incorporation was stopped by adding CHX (10 μg/mL). Cells were washed twice with FACS buffer, stained with fluorescently labeled anti‐IgM monovalent Fab fragments (Jackson ImmunoResearch), washed with FACS buffer and fixed for 30 min at room temperature with 1% paraformaldehyde (PFA) in FACS buffer. Samples were washed with FACS buffer and permeabilized with cytofix/cytoperm solution (BD Biosciences), for 20 min, at room temperature, while shaking. Samples were washed with 1X perm/wash buffer (BD Biosciences) and stained with anti‐Puromycin antibody (cl. 12D10‐A647, Millipore) 1 h at room temperature, while shaking. After washing with perm/wash buffer, samples were resuspended in PBS, acquired on a FACS Canto (BD Biosciences) and analyzed using FlowJo software (BD Biosciences).

### 2.7 Statistical Analyses

GraphPad Prism software v.10.1.1 was used for data visualization and statistical analyses. Depending on the experimental design, either unpaired Student's t tests or nonparametric Mann–Whitney tests were applied, as indicated. Drug screening data were analyzed in Matlab, as detailed in the corresponding section. Information on sample size, mean and median values, and the statistical tests used is provided in the Results section, figures, and figure legends. A *p* value < 0.05 was considered statistically significant for all analyses.

## 3 Results

### 3.1 Murine *λ*‐MYC Lymphomas Adapt to BCR Loss Without Losing Proliferative Potential

**FIGURE 1:**
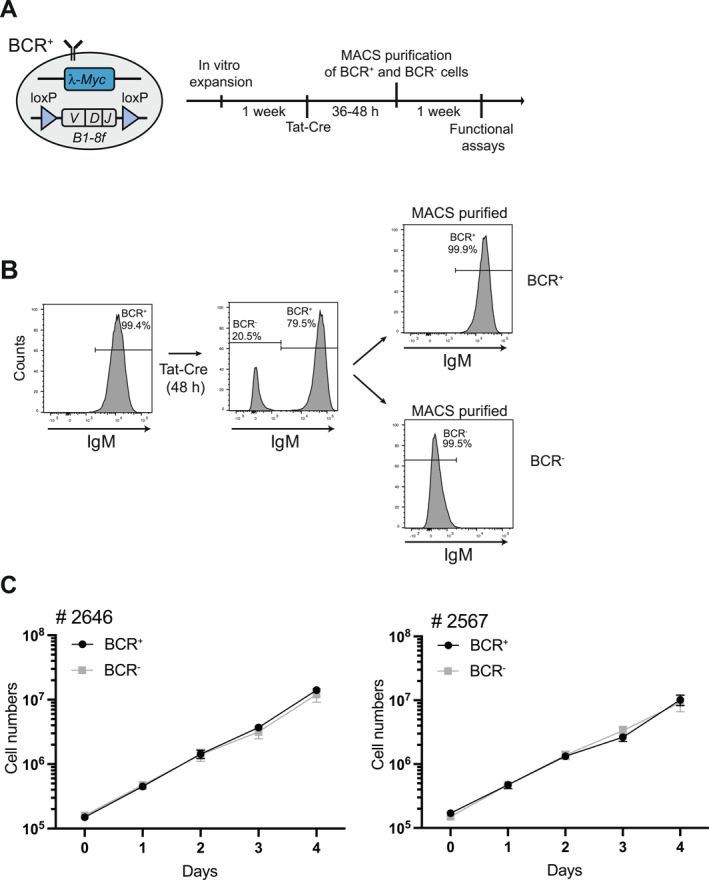
Murine *λ*‐MYC lymphomas adapt to inducible BCR extinction without losing proliferative capacity. (A) Cartoon depicting the genetic background of *λ*‐MYC; B1‐8f lymphomas and experimental workflow. (B) Flow cytometry analysis of representative (*n* = 3) *λ*‐MYC lymphoma (tumor # 2646) cultures prior to (−) and 48 h after (+) TAT‐Cre transduction, stained for surface IgM expression. Right histograms reveal the purity of BCR^+^ and BCR^−^ (flow through) *λ*‐MYC lymphomas isolated by magnetic cell sorting (MACS) using a monovalent anti‐IgM antibody. Numbers indicate frequency of cells. (C) Superimposition of representative cumulative growth curves of BCR^+^ and BCR^−^ *λ*‐MYC lymphoma cultures from two independent lymphomas (# 2646 and # 2567) grown in isolation for the indicated days. Cells were counted every 24 h. Data are representative of *n* = 5 (B), and *n* = 3 (C) replicates for each individual lymphoma line.

We previously reported the isolation of monoclonal primary B cell lymphomas from *λ*‐MYC transgenic mice carrying a pre‐rearranged immunoglobulin heavy (IgH) chain variable region gene (B1‐8f) flanked by loxP sites [13]. Cell lines established from two independent primary tumors were transiently transduced with cell‐permeable TAT‐Cre recombinant protein to delete the *B1‐8f* gene (Figure 1A). Flow cytometric analyses of cell suspensions 48 h post transduction revealed a distinct fraction of surface (s) IgM‐negative cells, confirming successful BCR inactivation in these cells (Figure 1B). IgM^+^ and IgM^−^ lymphoma cells were purified by magnetic cell sorting, cultured in isolation, and their growth was monitored over time. BCR^+^ and BCR^−^ cells showed comparable growth behavior, with an exponential growth rate shared by the two tumor subsets (Figure 1C). These results, which corroborate previous findings [13], highlight the ability of the majority of MYC‐transformed B cell lymphomas to rapidly adapt to the acute silencing of tonic antigen receptor signaling following inducible extinction of sBCR.

### 3.2 High‐Throughput Profiling Reveals Drug Sensitivities in Paired BCR^+^ and BCR^−^ *λ*‐MYC Lymphomas

**FIGURE 2:**
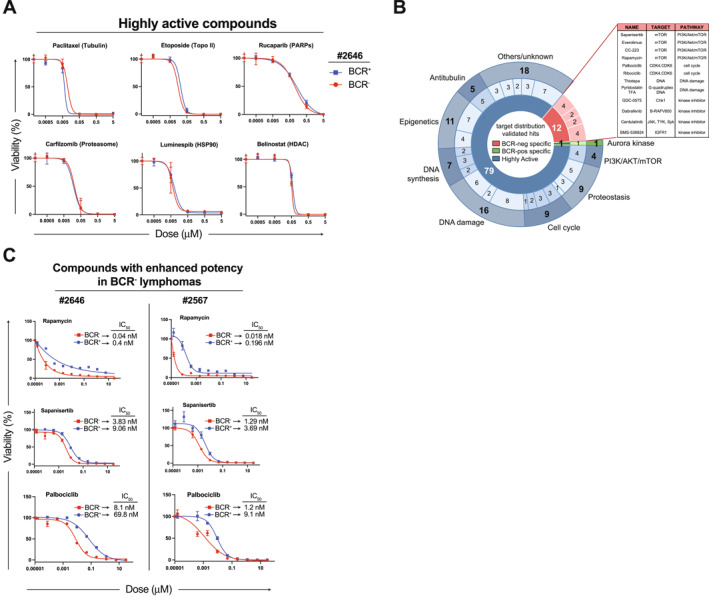
High‐throughput profiling reveals shared and differential drug sensitivity in paired BCR^+^ and BCR^−^ *λ*‐MYC lymphomas. (A) Dose‐response curves of representative drugs equally effective against BCR^+^ and BCR^−^ *λ*‐MYC lymphomas (IC_65_ < 0.5 μM). (B) Pie chart summarizing drug target categories that show high activity against MYC lymphoma B cells irrespective of BCR status (blue slices), and those displaying enhanced potency in BCR‐negative (red slices) or in BCR‐positive (green slice) lymphomas, grouped according to their mechanism of action or molecular target. Total number of compounds within each category (*n* = 79 highly active; *n* = 12 BCR^−^‐specific; *n* = 1 BCR^+^‐specific) is indicated in the inner tier. Numbers in the middle tier refer to the number of molecules hitting the same target. The number of compounds interfering with individual pathways is indicated in the outer tier. Compounds exhibiting preferential activity in BCR‐less lymphomas, together with their annotated targets and pathways, are listed in the in the pop‐up table. (C) Dose‐response curves of selected drugs (IC_65_ < 0.5 μM) showing enhanced potency against BCR^−^ cells in two independent lymphomas (# 2646, # 2567).

To identify vulnerabilities associated with loss of sBCR, we conducted a large‐scale pharmacological screen in matched BCR^+^ and BCR^−^ *λ*‐MYC lymphoma cells derived from the same parental tumor (2646). Cells were plated in 384‐well format and exposed to a drug library composed of 1475 small‐molecule compounds, including FDA‐approved agents, clinical candidates, and investigational molecules (Table S1). Each compound was tested across five concentrations (0.005–50 μM), and cell viability was assessed 24 h post‐treatment by ATP‐based luminescence assays. Cell‐viability values were normalized to vehicle‐treated controls. For each compound and dose, we calculated a differential viability metric (${\text{Viability}}_{{\text{BCR}}^{-}}$ ‐ ${\text{Viability}}_{{\text{BCR}}^{+}}$) and converted it into a z‐score using the median absolute deviation across the screen, enabling identification of compounds with BCR‐biased activity. Compounds achieving a z‐score ≤ −2 at one or more concentrations were classified as preferentially active in BCR^−^ cells, whereas those with a z‐score ≥ 2 were considered preferentially active in BCR ^+^ cells, and all candidates were further filtered by visual inspection of their dose–response curves to exclude aberrant profiles. In parallel, compounds that reduced viability of both BCR^+^ and BCR^−^ cells by ≥ 65% at the 0.5 μM dose were designated as highly active, capturing drugs with broad activity irrespective of BCR status. Primary screening revealed a broad spectrum of sensitivities (Table S1), with 141 compounds (9.5% of the library) meeting the ≥ 65% inhibition threshold at 0.5 μM in both BCR^+^ and BCR^−^ cells (Table S2). Based on primary screening performance, dose‐response patterns and target redundancy, 117 molecules (Table S3) were advanced to a validation screen based on either broad activity or evidence of BCR‐biased effects. Of these drugs, 83 agents (5.6% of the initial set) reproducibly fulfilled the ≥ 65% inhibition criterion in both populations, indicating that general vulnerabilities to DNA replication stress, microtubule damage, chromatin remodeling, and proteotoxic stress were retained upon BCR extinction (Figure 2A, Table S3). Conversely, 12 compounds consistently displayed preferential activity in BCR‐negative lymphoma cells, as defined by the z‐score thresholds and confirmed by manual inspection of their dose‐response curves (Figure 2B, Table S3 and Supporting Information S1: Figure S1). BCR^−^‐biased drugs included multiple inhibitors of mTOR and cyclin‐dependent kinases 4/6 (CDK4/6), revealing increased dependency of BCR‐less lymphomas on these pathways. Follow‐up dose‐response assays in two independent lymphomas (# 2646 and # 2567) confirmed preferential sensitivity of BCR^−^ cells to mTOR inhibitors (Sapanisertib and Rapamycin) and the CDK4/6 inhibitor Palbociclib (Figure 2C), with IC_50_ values in the nano‐to sub‐nanomolar range. A single compound showed higher potency in BCR^+^ cells and is reported in Table S2. Together, these results delineate a broad spectrum of pharmacological responses to BCR loss and identify candidate compounds whose efficacy is either maintained or potentiated in MYC‐driven lymphomas adapting to BCR extinction.

### 3.3 BCR Extinction Weakens mTOR‐Controlled Protein Synthesis and Sensitizes MYC Lymphomas to mTOR Inhibition

**FIGURE 3:**
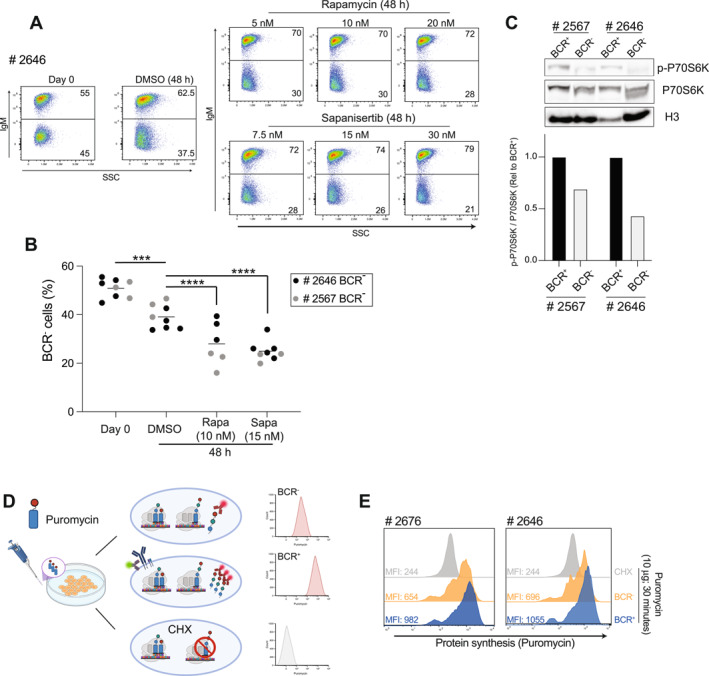
BCR extinction weakens mTOR‐regulated protein synthesis and sensitizes MYC lymphomas to mTOR inhibition. (A) Representative flow cytometric analyses of 1:1 mixture of BCR^+^ and BCR^−^ cells (# 2646) co‐cultured for 48 h with increasing doses of mTOR inhibitors Rapamycin or Sapanisertib. Vehicle (DMSO) treated cells served as control. (B) Quantification of BCR^−^ cell frequency in co‐cultures described in (A) at baseline (day 0) and after 48 h of treatment with Rapamycin (10 nM), Sapanisertib (15 nM), or DMSO. Black and grey circles indicate values from # 2646 and # 2567 lymphomas, respectively. (C) Immunoblot analysis of P70S6K phosphorylation in paired BCR^+^ and BCR^−^ lymphoma cells derived from # 2567 and # 2646 tumors. Histograms show phospho‐P70S6K levels normalized to total P70S6K and expressed relative to the matched BCR^+^ cells, as quantified from the immunoblot shown above. Data are representative of four independent biological replicates corresponding to distinct lymphoma lines (# 2567, # 2646, # 2564, and # 2676). (D) Experimental workflow for quantifying de novo protein synthesis in *λ*‐MYC lymphomas using the SUnSET assay. Lymphoma cells were shortly pulsed with puromycin, followed by flow‐cytometric detection of puromycin incorporation into nascent polypeptides in IgM^+^ and IgM^−^ cells. Cycloheximide‐treated cells were included as negative controls to verify suppression of puromycin incorporation. (E) Histogram representation of intracellular puromycin incorporation, measured by flow cytometry, in BCR^+^ (blue) and BCR^−^ (orange) cultures derived from two independent *λ*‐MYC lymphomas (# 2646 and # 2676). Cells were shortly pulsed with puromycin (10 μg/mL), and incorporation into nascent polypeptides quantified as median fluorescence intensity (MFI). Cycloheximide‐treated cells (grey), acted as negative controls. Data are representative of *n* = 3 (A), *n* = 3 (Rapa) and *n* = 5 (Sapa) (B) and *n* = 2 (C, E) independent experiments for each lymphoma line. Statistical comparisons in panel B were performed using a two‐way ANOVA (***p < 0.001; ****p < 0.0001).

Results from the drug‐screening indicated increased susceptibility of BCR‐deficient lymphomas to inhibitors targeting the mTORC1/2 complexes. To validate and mechanistically interpret this finding, we performed competitive co‐culture assays in which equal numbers of BCR^+^ and BCR^−^ cells from two independent *λ*‐MYC tumors (# 2646, # 2567) were mixed at a 1:1 ratio and treated for 48 h with mTORi Rapamycin or Sapanisertib, at sub‐lethal concentrations. Flow cytometric monitoring of lymphoma co‐cultures before and after treatment showed that, although both BCR^+^ and BCR^−^ cells were affected by mTOR inhibition, the proportion of BCR^−^ cells consistently declined, indicating greater vulnerability (Figure 3A–B, Supporting Information S1: Figure S2A). To examine whether mTOR signaling was influenced by BCR status, we assessed the extent of S6 kinase phosphorylation (p‐P70S6K) by immunoblotting of lysates isolated from matched BCR^+^ and BCR^−^ cultures from two independent lymphomas (# 2646, # 2567). Densitometric quantification showed consistently reduced p‐P70S6K levels in BCR‐deficient cells, despite comparable total P70S6K protein levels (Figure 3C), pointing to diminished mTOR pathway activation. Given the role of mTOR in controlling protein synthesis, we next quantified nascent polypeptide production using the SUnSET assay. After a short puromycin pulse of BCR^+^ and BCR^−^ lymphoma cells in vitro, incorporation of puromycin into newly synthesized proteins was measured by flow cytometry. BCR‐negative tumor cells incorporated less puromycin than their BCR‐positive counterparts (Figure 3D–E), indicating reduced rates of de novo protein synthesis.

Collectively, these findings show that loss of BCR expression weakens mTOR‐dependent anabolic activity in MYC lymphomas, resulting in lower translational output and heightened vulnerability to mTOR inhibition.

### 3.4 BCR Loss Reduces Cyclin D3 Levels and Heightens Lymphoma Sensitivity to CDK4/6 Inhibition

**FIGURE 4:**
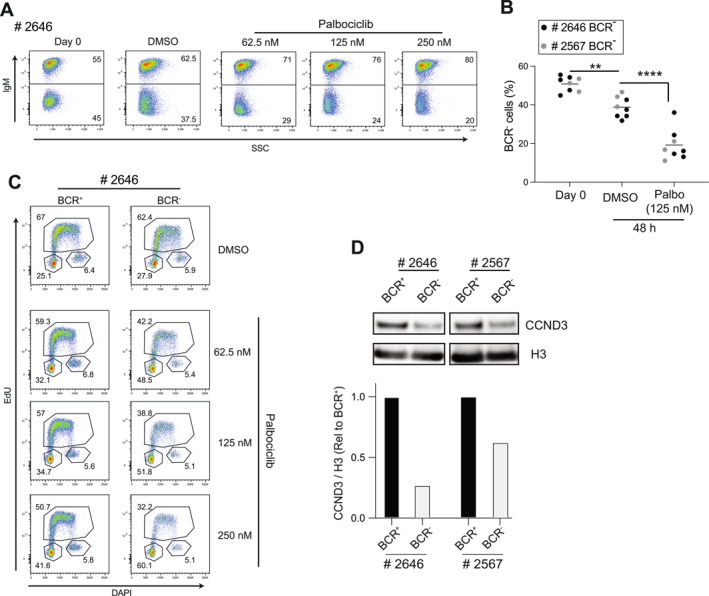
BCR loss reduces Cyclin D3 levels and heightens lymphoma sensitivity to CDK4/6 inhibition. (A) Representative flow‐cytometric analyses of *λ*‐MYC lymphoma cells (# 2646) assessing the frequency of BCR^+^ and BCR^−^ subsets co‐cultured at a 50:50 starting ratio and treated for 48 h with increasing doses of Palbociclib. Vehicle (DMSO)–treated cultures served as controls. The starting distribution of BCR^+^ and BCR^−^ cells (day 0) is shown on the left. (B) Quantification of BCR^−^ cell frequency in co‐cultures described in (A) at baseline (day 0) and after 48 h of treatment with Palbociclib (125 nM). Black and grey circles indicate values obtained from lymphomas # 2646 and # 2567, respectively. (C) Cell‐cycle analysis of BCR^+^ and BCR^−^ cells from mixed cultures of *λ*‐MYC lymphoma # 2646 following 48 h of treatment with the indicated doses of Palbociclib. Vehicle (DMSO)‐treated cells were used as reference. (D) Immunoblot analysis of Cyclin D3 (CCND3) protein levels in paired BCR^+^ and BCR^−^ lymphoma cells derived from two independent tumors (# 2646 and # 2567). The corresponding densitometric quantification of CCND3 normalized to protein input is reported in the histogram. Data are representative of *n* = 3 (A), *n* = 5 (B), *n* = 3 (C), and *n* = 3 (D) independent experiments. Statistical comparisons in panel B were performed using a two‐way ANOVA (**p < 0.01; ****p < 0.0001).

Among drug categories displaying enhanced potency in BCR‐deficient lymphomas, CDK4/6 inhibitors ranked prominently. To validate this result, competitive growth assays were performed using paired BCR^+^ and BCR^−^ cells from two independent *λ*‐MYC lymphomas (# 2646, # 2567) co‐cultured at equal ratios and exposed to sub‐lethal doses of Palbociclib for 48 h. Flow‐cytometric quantification revealed a consistent decline in the frequency of BCR^−^ cells relative to their BCR^+^ counterparts (Figure 4A–B, Supporting Information S1: Figure S3A), indicating that BCR‐deficient cells are more affected by CDK4/6 inhibition. Cell‐cycle analysis by DNA‐content profiling in Palbociclib‐treated cells showed a more pronounced accumulation of BCR‐deficient lymphomas in the G_0_/G_1_ phase of the cell‐cycle, consistent with a stronger G_1_‐to‐S arrest imposed by CDK4/6 blockade (Figure 4C, Supporting Information S1: Figure S3B). To investigate the molecular basis of this differential response, we compared the abundance of CDK4 and CDK6 across matched BCR^+^ and BCR^−^ lymphoma variants. Immunoblot analyses revealed subtle changes in CDK4 and CDK6 protein levels following BCR extinction (Supporting Information S1: Figure S3C and data not shown), indicating that altered CDK expression does not account for the increased sensitivity to Palbociclib. We therefore hypothesized that, upon BCR loss, enhanced Glycogen Synthase Kinase‐3‐beta (GSK3β) activity [13] might accelerate the degradation of its established substrate Cyclin D3 (CCND3), a key regulator of CDK4/6 in promoting G_1_‐to‐S cell‐cycle progression. Consistent with this model, immunoblotting analyses revealed reduced CCND3 protein levels in BCR‐negative *λ*‐MYC lymphoma cells from two independent tumors compared with their paired BCR^+^ counterparts (Figure 4D). These findings suggest that reduced availability of CDK4/6‐CCND3 complexes sensitizes BCR‐deficient cells to CDK4/6 inhibition, thereby reinforcing the G_1_‐S cell‐cycle blockade.

Together, these results establish a mechanistic link between BCR loss, diminished CCND3 levels, and increased susceptibility to CDK4/6 inhibitors, delineating a targetable vulnerability in MYC‐driven lymphomas resisting BCR extinction.

### 3.5 BCR‐Null Human HGBCL With *MYC* and *BCL2* Rearrangements Display Marked Sensitivity to CDK4/6 and mTOR Inhibition

**FIGURE 5:**
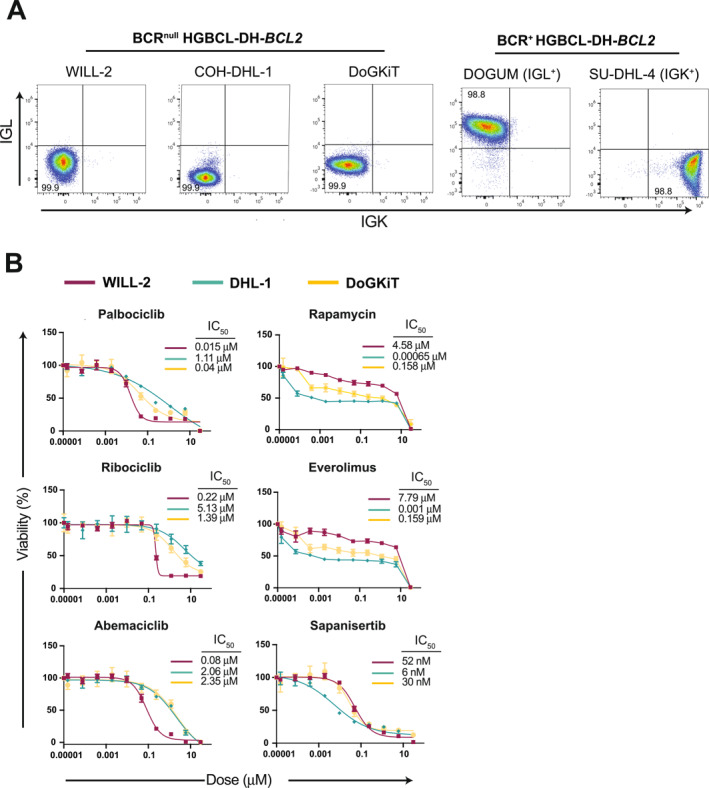
BCR‐null human HGBCL with *MYC* and *BCL2* rearrangements are highly sensitive to CDK4/6 and mTOR inhibition. (A) Flow‐cytometric analysis of surface IG‐Kappa (IGK) and IG‐Lambda (IGL) expression in human BCR‐null HGBCL‐*DH*‐*BCL2* cell lines (WILL‐2, COH‐DHL1, and DoGKIT). IGK^+^ (SU‐DHL‐4) and IGL^+^ (DOGUM) B lymphoma cell lines were included as reference controls. (B) Dose–response curves and IC_50_ values for clinically approved CDK4/6 inhibitors (Palbociclib, Ribociclib, Abemaciclib) and mTOR inhibitors (Rapamycin, Everolimus, Sapanisertib) following 72 h treatment of the indicated human BCR‐null HGBCL‐DH‐*BCL2* cell lines.

To validate our findings in human MYC‐driven lymphomas, we analyzed the human HGBCL‐DH‐*BCL2* cell line models WILL‐2, DoGKIT, and COH‐DHL1, which have spontaneously silenced BCR expression, as determined by flow‐cytometric staining for surface IG light chain expression. IG‐Kappa (SU‐DHL4) and IG‐Lambda (DOGUM) expressing lymphoma lines acted as positive control for sBCR expression (Figure 5A). Cells were treated with increasing concentrations of clinically approved CDK4/6 inhibitors (Palbociclib, Ribociclib and Abemaciclib), or mTOR inhibitors (Rapamycin, Everolimus and Sapanisertib), and IC_50_ values were determined after 72 h. Extending our previous analyses [11], HGBCL‐DH‐*BCL2* models consistently displayed pronounced sensitivity to both drug classes (Figure 5B). The relative potency mirrored the pattern observed in murine *λ*‐MYC lymphomas, supporting conservation of the underlying vulnerabilities.

These results validate the translational relevance of the murine findings and highlight CDK4/6 and mTOR pathways as therapeutically actionable dependencies in human HGBCL‐DH*‐BCL2* and possibly other MYC‐driven aggressive B cell lymphomas that have silenced BCR expression.

## 4 Discussion

This study identifies actionable vulnerabilities that arise in MYC‐driven B cell lymphomas experiencing inducible BCR extinction. By conditionally inactivating expression of the IG heavy chain in the *λ*‐MYC lymphoma model rendering cells BCR deficient, and performing a large‐scale pharmacological screen, we uncovered a subset of compounds, including clinically approved agents, that retain or gain efficacy in the BCR‐less state. These findings extend and complement previous observations in both mouse and human lymphoma models [13, 14] showing that MYC‐driven B cell lymphomas can sustain proliferation despite abrupt BCR extinction, yet undergo metabolic and cell‐cycle adaptations that generate new therapeutic dependencies.

Mechanistically, loss of BCR signaling attenuates PI3K‐mTOR activity [13], leading to reduced phosphorylation of P70S6K and impaired global protein synthesis, consistent with partial restriction of anabolic metabolism. In parallel, in *λ*‐MYC lymphomas, the weakening of AKT‐mediated GSK3β inhibition [13] enhances GSK3β‐dependent phosphorylation and degradation of CCND3, thereby diminishing the pool of CDK4/6‐CCND3 complexes required for promoting G1‐to‐S cell‐cycle progression. This dual constraint on protein synthesis and cell‐cycle control renders BCR‐deficient lymphomas hypersensitive to pharmacologic inhibition of the mTOR and CDK4/6 pathways. Collectively, these adaptive responses illustrate the remarkable plasticity of MYC‐driven lymphoma B cells in tolerating the loss of BCR expression, while at the same time exposing vulnerabilities that can be leveraged therapeutically.

The translational implications of these findings are particularly relevant in the context of recently approved frontline regimens such as pola‐R‐CHP, which incorporate polatuzumab vedotin for selective cytotoxic delivery to DLBCL and HGBCL [7, 9]. While these therapies rely on sustained surface BCR expression for effective drug delivery, the spontaneous emergence of BCR‐negative variants among MYC‐driven lymphomas represents a plausible mechanism of primary or acquired resistance [11, 17]. Our results suggest that pharmacologic inhibition of the mTOR and CDK4/6 pathways, along with other targets emerging from the screening, could be incorporated into second line treatments to eliminate BCR‐negative subclones arising after CD79B‐directed therapy. Alternatively, upfront co‐administration of polatuzumab vedotin with these inhibitors could pre‐empt resistance by limiting the emergence and expansion of BCR‐silenced variants. This strategy aligns with recent findings [17], showing that disulfiram can potentiate polatuzumab vedotin activity in lymphoma models with poor intrinsic responsiveness to the ADC, underscoring the broader therapeutic value of rational ADC‐based combination approaches. Previous clinical attempts to use mTOR or CDK4/6 inhibitors in aggressive B cell lymphomas [18, 19, 20, 21] have reported objective responses but overall modest and short‐lived activity in small and biologically unselected cohorts, which has so far precluded their routine integration into DLBCL or HGBCL management. By introducing BCR status as a key biological variable, our data suggest that BCR‐silenced MYC‐expressing GCB‐DLBCL and HGBCL [11] could constitute enriched patient subsets in which mTOR‐ and CDK4/6‐directed strategies achieve deeper and more durable benefit. In this view, prospective diagnostic assessment of BCR/CD79 B expression and complex formation at diagnosis and eventually at relapse, may improve patient selection for regimens that combine CD79B‐targeted ADCs with inhibitors of mTOR or CDK4/6. Although diagnosis and response assessment currently rely on tissue biopsies, emerging liquid biopsy strategies may ultimately allow longitudinal detection of BCR‐silenced malignant clones during disease evolution and treatment [22]. Such approaches could include tracking the emergence of *IGV* gene crippled subclones, or evidence of ongoing IG light chain editing [11], as well as monitoring recurrent gain‐of‐function mutations in genes of the PI3K‐AKT and RAS‐MAPK pathways previously associated with antigen‐receptor independence in aggressive B‐cell lymphomas [11, 13]. Notably, both mTOR and CDK4/6 inhibitors are already clinically approved and display manageable toxicity profiles. At the same time, optimal scheduling, dose intensity, and treatment duration will need to be carefully defined before such combinations can be translated clinically into existing regimens for aggressive B cell lymphomas.

Our study also provides a framework for understanding how MYC‐driven lymphomas adapt to the loss of receptor‐dependent trophic signals. The observation that BCR‐negative cells maintain proliferation yet exhibit reduced anabolic capacity implies a partial uncoupling of MYC transcriptional output from mTOR‐mediated translation. This decoupling may allow survival under nutrient limitation or therapeutic stress but simultaneously creates dependency on residual anabolic control. It will be important to determine whether these adaptive features extend to other mature B cell lymphoma subtypes in which BCR signaling is bypassed, including BCR‐less variants of Burkitt lymphoma, GCB‐type DLBCL, and HGBCL [11, 13], and whether similar adaptive states arise in vivo under therapeutic pressure from CD79B‐targeted ADCs. We acknowledge that our study does not discriminate between rapid signaling rewiring in BCR‐less lymphomas cells and the preferential outgrowth of pre‐existing subclones better suited to survive BCR extinction. More refined lineage‐tracing and single‐cell approaches will be required to resolve their relative impact.

Another limitation of this work is that the primary pharmacological screen was conducted in matched BCR‐positive and BCR‐negative derivatives from a single parental *λ*‐MYC; B1‐8f tumor line. Although key dependencies were validated in two independent murine lymphoma lines and in multiple human HGBCL‐DH‐*BCL2* cell line models that have spontaneously silenced BCR expression, inter‐tumoral heterogeneity may influence the spectrum and magnitude of BCR extinction‐associated drug vulnerabilities, and will require future studies in additional genetic backgrounds and in vivo models.

In summary, this study provides the first systematic assessment of drug vulnerabilities in MYC‐driven mature B cell lymphomas adapting to acquired BCR extinction. Through this approach, we identified heightened sensitivity of BCR‐deficient lymphomas to inhibition of the mTORC1/2 and CDK4/6‐CCND3 pathways, revealing targetable dependencies that become more pronounced upon loss of BCR signaling. These findings reveal how metabolic and cell‐cycle adaptations, while enabling continued proliferation without tonic BCR signaling, simultaneously expose weaknesses that can be therapeutically exploited. By integrating these adaptive vulnerabilities into treatment design, our work offers a rationale for combining or sequentially aligning CD79B‐directed therapies with agents targeting the mTOR and CDK4/6 pathways to counteract resistance and improve outcomes in aggressive MYC‐driven B cell lymphomas.

## Author Contributions

S.B., N.C.P., A.P., A.A., H.A., G.F., B.L., L.P., M.R., M.P., F.M. and D.F. performed experiments. S.B., N.C.P, A.P., G.V., C.M. and S.C. analyzed and interpreted the data. S.C., C.M and G.V. acquired fundings. S.C., G.V., C.M. and S.B. wrote the manuscript. S.C., G.V., C.M, and M.V. supervised different stages of the project. All authors reviewed and approved the final version of the manuscript.

## Funding

This work was supported by grants from the Italian Association for Cancer Research (AIRC; IG #23747 to S.C. and AIRC; IG #21328 to C.M.). Through S. Casola, this work has been supported by the project SCALE UP—Department of Excellence 2023–2027, funded by the Italian Ministry of University and Research to the Department of Molecular Biotechnology and Translational Medicine, University of Milan. S.C. acknowledges funding from the SMART‐FL project by the NRRP Next Generation EU—Mission 4, Component 2, Investment 1.4—Project CN00000041 “National Center for Gene Therapy and Drugs based on RNA Technology”—CUP B83C22002870006. G.V. received support from the American Society of Hematology Global Research Award (AGRA2023‐4) and the Marie Skłodowska‐Curie postdoctoral training program (H2020‐MSCA‐IF‐2019 #895887). The Experimental Therapeutics Program was also supported by generous donation from Ravelli family.

## Ethics Statement

The authors have nothing to report.

## Conflicts of Interest

The authors declare no conflicts of interest.

## Supporting information


Supporting Information S1

**Figure S1:** Compounds excluded after the validation run.

**Figure S2:** BCR extinction weakens mTOR–driven protein synthesis and sensitizes MYC lymphomas to mTOR inhibition.

**Figure S3:** BCR loss increases lymphoma sensitivity to CDK4/6 inhibition.

**Table S1:** Set of compounds evaluated in the primary screening.

**Table S2:** Set of compounds showing a percent of viability < 35% in both e BCR negative and positive cells at 0.5 uM.

**Table S3:** List of compounds selected for the validation screening.

**Table S4:** Key resources.

## Data Availability

The authors have nothing to report.
