# Supplementary material for: Targetable Vulnerabilities in MYC‐Driven B Cell Lymphomas Resistant to BCR Extinction
Source: Hematol Oncol. 2026 Feb 11;44(2):e70175. doi: 10.1002/hon.70175 (PMC12892111; doi:10.1002/hon.70175)
Supplement: Supplementary file 1 — Supporting Information S1 [file HON-44-e70175-s004.docx]

**SUPPLEMENTARY INFORMATION**

**Legends to Supplementary Figures and Tables**

**Supplementary Figure 1 (related to Figure 2)**: **Compounds excluded after the validation run.** Dose-response curves for the compounds showing z-scores of differential viability < -2 or > +2 (upper and lower panels, respectively) that were excluded from the final selection following visual inspection, due to high variability at the dose meeting the selection criteria.

**Supplementary Figure 2 (related to Figure 3)**: **BCR extinction weakens mTOR-driven protein synthesis and sensitizes MYC lymphomas to mTOR inhibition**. (**A**) Representative flow-cytometric analyses of # 2567 λ-MYC lymphoma cells to assess the frequency of BCR⁺ and BCR⁻ cells in 50:50 co-culture assays treated for 48 h with increasing doses of the mTOR inhibitors Rapamycin or Sapanisertib. Vehicle (DMSO)-treated cells served as control. Frequencies of BCR⁺ and BCR⁻ cells at the start of the assay (day 0) are shown on the left. Data are representative of n = 3 independent experiments.

**Supplementary Figure 3 (related to Figure 4): BCR loss increases lymphoma sensitivity to CDK4/6 inhibition**. (**A)** Frequency of BCR⁺ and BCR⁻ cells from the λ-MYC lymphoma # 2567 co-cultured at a 50:50 starting ratio and treated for 48 h to increasing doses of Palbociclib, as determined by flow cytometric analysis. Vehicle (DMSO)-treated cells were used as controls. Frequencies of BCR⁺ and BCR⁻ cells at the start of the co-culture (Day 0) are shown in the left plot. (**B**) Cell-cycle analysis of BCR⁺ and BCR⁻ cells (# 2567) from ~50:50 mixed cultures after 48 h of treatment with the indicated concentrations of Palbociclib, assessed by flow cytometry. Vehicle (DMSO)-treated cells served as reference. (**C**) Immunoblot analysis of CDK4 protein levels in paired BCR⁺ and BCR⁻ tumor cells grown in isolation, derived from λ-MYC lymphomas # 2646 and # 2567. Numbers indicate CDK4 levels normalized to protein input.

**Supplementary Table 1**. **Set of compounds evaluated in the primary screening.** List of compounds included in the primary screening, classified according to name, annotated target(s), and pathway classification. Percent of BCR-negative and BCR^+^ viable cells (%), relative to untreated control, achieved at 0,5 μM dose, for each compound used in the screening is reported.

**Supplementary Table 2. Set of compounds showing a percent of viability < 35% in both e BCR negative and positive cells at 0.5 μM**.

**Supplementary Table 3**. **List of compounds selected for the validation screening.** Subset of compounds advanced from the primary screen to the validation screen. Compounds are classified according to the criteria defined at the top of the table as highly active in BCR⁺ and BCR-negative lymphomas, or as showing preferentially enhanced potency in BCR^+^ or BCR-less cells. A subset of compounds failed to reproduce the effects observed in the primary screen and did not meet any of the predefined classification criteria (not classified). For each compound, name, annotated target(s), pathway classification, and outcome of the validation screening are reported. Cell viability (%) of BCR-negative and BCR^+^ lymphoma cells relative to untreated control, is shown upon treatment with scalar drug concentrations**;** the *z-score* of differential viability between BCR^-^ and BCR⁺ is reported. IC₅₀ values calculated for BCR⁺ and BCR⁻ lymphoma cells are provided to enable direct comparison of compound potency across the two cell populations.

**Supplementary Table 4**. **Key resource table.** List of reagents used in the study.

**Supplementary Methods and Materials**

Lymphoma growth curve analysis

Growth curves were generated using IgM⁺ and IgM⁻ lymphoma B cells obtained by MACS purification following acute Tat-Cre transduction. Purified cells were cultured only briefly prior to the assay to minimize adaptation to BCR loss. For each lymphoma line, triplicate cultures of BCR⁺ and BCR-less cells were established and seeded at 1x10⁵ cells/mL in complete medium, then maintained in isolation for 5 days. Cultures were split every 48 hours to preserve cell densities within the exponential growth range. Cell numbers were quantified every 24 hours using a haemocytometer, and the mean of at least two independent counts per replicate was recorded. Cumulative cell growth was calculated by correcting cell numbers for the dilution factor introduced at each splitting time.

Immunoblotting analysis

Immunoblotting was performed according to standard procedures. Cells were lysed in RIPA buffer (50 mM Tris-HCl, pH 7.4; 5 mM MgCl₂; 150 mM NaCl; 1% Triton X-100) supplemented with protease and phosphatase inhibitors (Roche). Protein lysates were denatured in Bolt LDS sample buffer (Thermo Fisher Scientific) containing Bolt reducing agent and resolved on Novex Bolt 4–12% Bis-Tris gels (Life Technologies/Thermo Fisher Scientific). Proteins were transferred to nitrocellulose membranes (iBlot™ 2 Transfer Stacks) using the iBlot™ 2 Gel Transfer Device. Membranes were blocked with 5% milk in TBS-T (0.1% Tween-20) for 1 h at room temperature and incubated overnight at 4°C with primary antibodies (listed in Supplementary Table 3) diluted in 5% BSA in TBS-T. After three washes in TBS-T, membranes were incubated with HRP-conjugated secondary antibodies (Bio-Rad Laboratories / Cell Signaling Technology) and developed using Dura ECL substrate (Thermo Fisher Scientific). Chemiluminescent signals were acquired with the ChemiDoc MP Imaging System (Bio-Rad Laboratories). Raw images were analyzed using ImageLab software (Bio-Rad Laboratories). Expression of individual protein was normalized to protein input using ImageLab software (Bio-Rad Laboratories).

Flow cytometric analysis and cell sorting

Lymphoma B cells were washed in PBS and stained in FACS buffer (1% BSA, 2 mM EDTA, 0.01% sodium azide in PBS) with combinations of fluorescent-labelled antibodies (listed in Supplementary Table 3) for 20 min on ice, protected from light. After staining, cells were washed twice with FACS buffer and analyzed on a Cytek Aurora spectral analyzer (Cytek Biosciences). BCR-negative lymphoma cells were isolated using a FACS Aria cell sorter (BD Biosciences) in sorting medium (DMEM supplemented with 30% FBS, 2 mmol/L L-glutamine, and 1× penicillin/streptomycin). Dead cells were excluded using propidium iodide and/or standard physical parameters. Flow cytometry data were analyzed using FACSDiva (BD Biosciences), SpectroFlo (Cytek Biosciences), and FlowJo software (v10.9.0, BD Biosciences).

Cell-cycle analysis

Lymphoma B-cell cultures were pulsed with EdU (10 µM, Invitrogen) for 12 minutes, followed by EdU detection. EdU-pulsed cells were harvested and stained with fluorescently labelled anti-IgM monovalent Fab fragments (Jackson ImmunoResearch, Supplementary Table 3). After washing in 1% BSA in PBS, cells were resuspended in 100 µL of 1% PFA in PBS and fixed for 15 minutes at room temperature, protected from light. Cells were then washed and permeabilized for 25 minutes in 50 µL of 1% BSA in PBS supplemented with 0.5% saponin. EdU was detected by incubating the cells for 5 minutes with a TBS-based reaction cocktail containing CuSO₄ (4 mM), sodium ascorbate (100 mM), and Sulfo-Cyanine5 azide (2 µM), protected from light. Following washes with saponin-based permeabilization and wash (PW) buffer, cells were resuspended in 100 µL of PW buffer supplemented with RNase A (0.5 mg/mL) and DAPI (2.5 µg/mL) for DNA-content staining. Samples were acquired on a FACS Canto (BD Biosciences) or Cytek Aurora spectral analyzer (Cytek Biosciences) and analyzed using FlowJo software (v10.9.0, BD Biosciences).
