# Supplementary material for: Targetable Vulnerabilities in MYC‐Driven B Cell Lymphomas Resistant to BCR Extinction
Source: Hematol Oncol. 2026 Feb 11;44(2):e70175. doi: 10.1002/hon.70175 (PMC12892111; doi:10.1002/hon.70175)

Supplementary Figure 1 - related to Figure 2

Compounds with z-scores < -2 that failed visual inspection

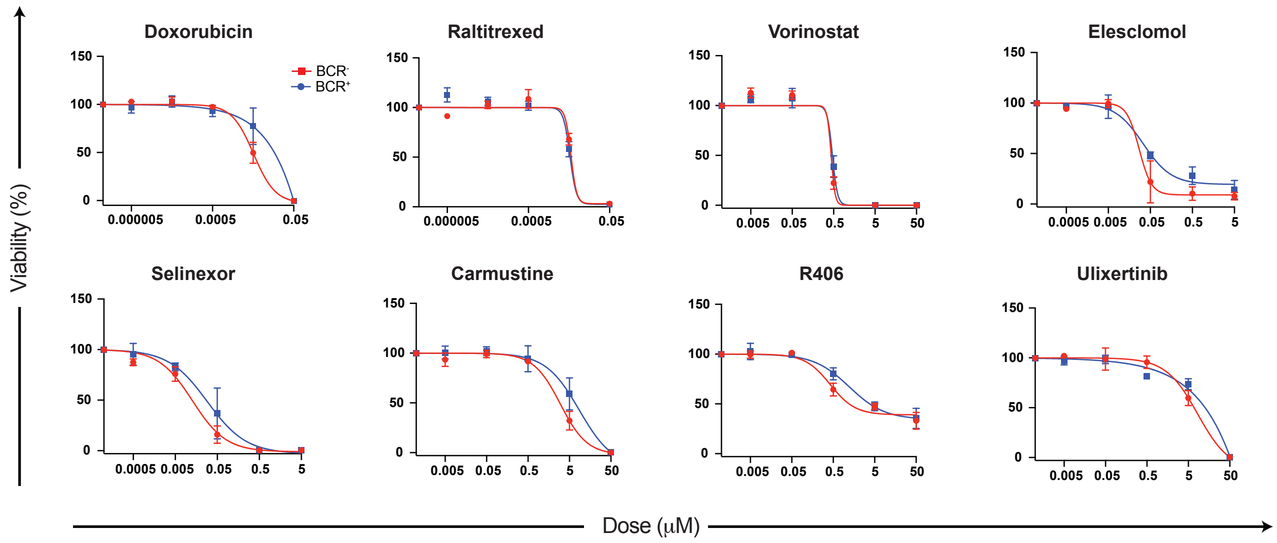

Compounds with z-scores > +2 that failed visual inspection

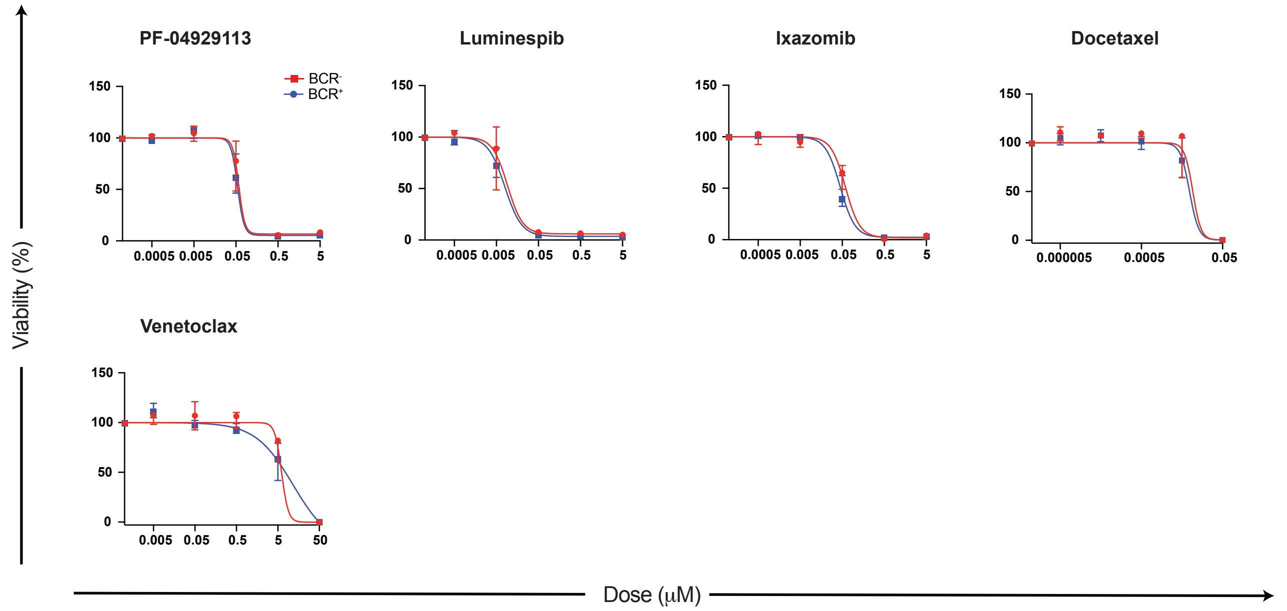

Supplement: Supplementary file 2 — Figure S1: Compounds excluded after the validation run. [file HON-44-e70175-s001.pdf]
