# Supplementary figures and images for: Targetable Vulnerabilities in MYC‐Driven B Cell Lymphomas Resistant to BCR Extinction
Source: Hematol Oncol. 2026 Feb 11;44(2):e70175. doi: 10.1002/hon.70175 (PMC12892111; doi:10.1002/hon.70175)

Supplementary Figure 2 - related to Figure 3

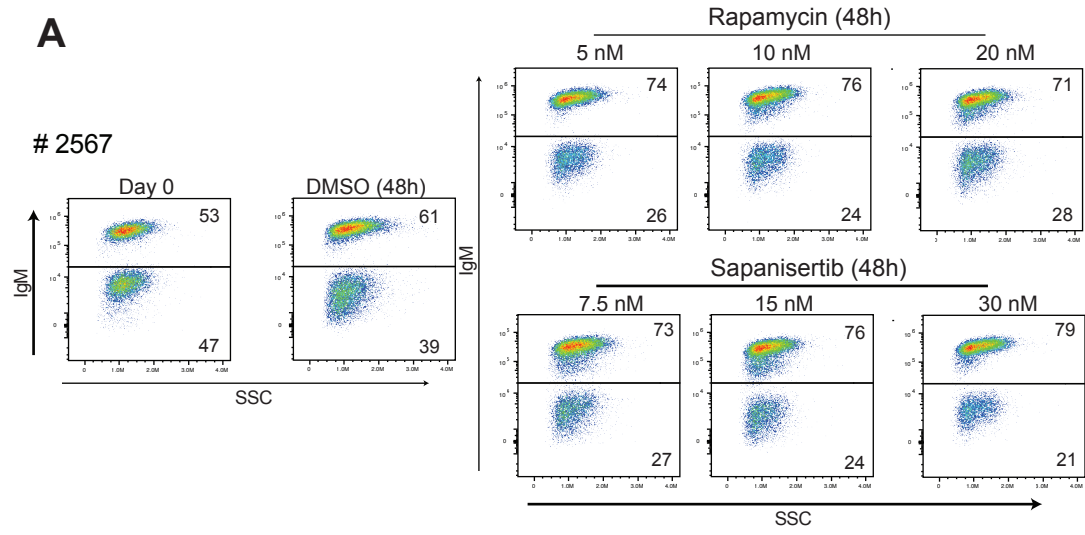

Supplement: Supplementary file 3 — Figure S2: BCR extinction weakens mTOR–driven protein synthesis and sensitizes MYC lymphomas to mTOR inhibition. [file HON-44-e70175-s002.pdf]

Supplementary Figure 3 - related to Figure 4

A

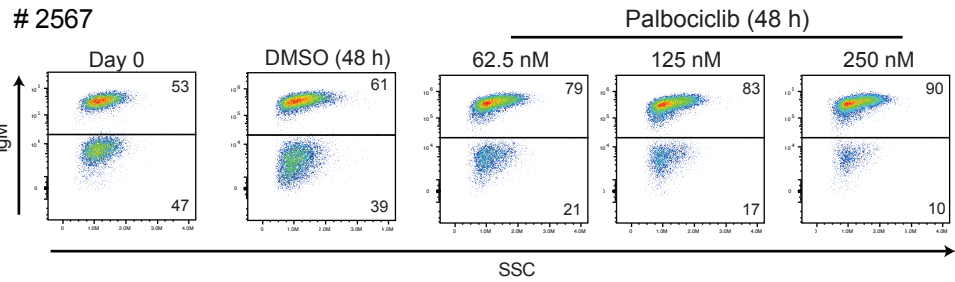

B

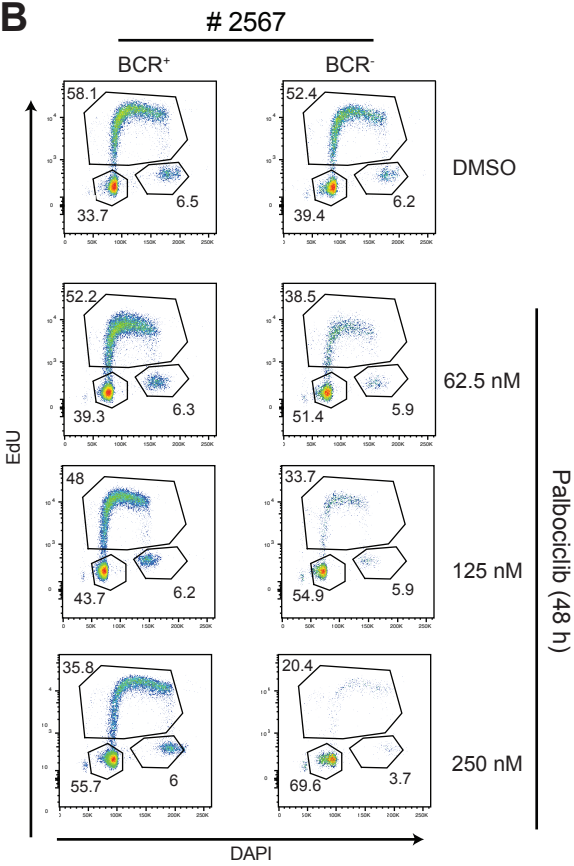

C

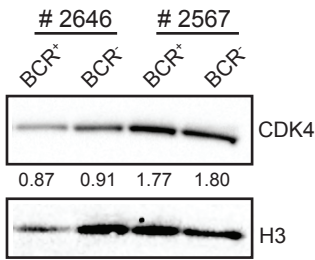

Supplement: Supplementary file 4 — Figure S3: BCR loss increases lymphoma sensitivity to CDK4/6 inhibition. [file HON-44-e70175-s006.pdf]
